# The role of hydrogen-bonded interphase in achieving optimal performance of nitrile-butadiene rubber/graphene oxide nanocomposites

**DOI:** 10.3389/fchem.2025.1710575

**Published:** 2026-01-02

**Authors:** Talia Tene, Lala Gahramanli, Mustafa Muradov, Aynur Mammadova, Vugar Khudaverdiev, Aida Azizova, Shafiga Alakbarova, Lala Isayeva, Rashida Huseynzade, Goncha Eyvazova, Flora Hajiyeva, Stefano Bellucci, Cristian Vacacela Gomez, Haji Vahid Akhundzada, Rana Khankishiyeva

**Affiliations:** 1 Department of Chemistry, Universidad Técnica Particular de Loja, Loja, Ecuador; 2 Nano Research Laboratory, Center of Excellence, Baku State University, Baku, Azerbaijan; 3 Faculty of Physics, Chemical Physics of Nanomaterials, Baku State University, Baku, Azerbaijan; 4 Technology of Organic Substances and High Molecular Compounds, Azerbaijan State Oil and Industry University, Baku, Azerbaijan; 5 Institute of Radiation Problems, Ministry of Science and Education of the Republic of Azerbaijan, Baku, Azerbaijan; 6 NAA – National Aviation Academy, Baku, Azerbaijan; 7 National Institute of Materials Physics, Magurele, Romania; 8 Department of Environmental Engineering (DIAm), University of Calabria, Rende, Italy; 9 Universidad Ecotec, Samborondón, Ecuador; 10 ICRL Industrial Chemistry Research Laboratory, Baku State University, Baku, Azerbaijan; 11 Scientific-Research Institute ― Geotechnological Problems of Oil, Gas, and Chemistry, Azerbaijan State Oil and Industry University, Baku, Azerbaijan; 12 Azerbaijan University of Architecture and Construction, Baku, Azerbaijan

**Keywords:** nitrile-butadiene rubber, graphene oxide, rubber nanocomposites, filler dispersion, mechanical properties, thermal stability, interfacial interaction, agglomeration

## Abstract

Graphene oxide (GO) nanosheets (0.5–2.0 phr) were incorporated into nitrile-butadiene rubber (NBR) to clarify how interfacial chemistry and dispersion control macroscopic performance. GO was synthesized by a modified Hummers method, and different filler concentrations of NBR/GO were prepared via solution–coagulation followed by sulfur vulcanization. Transmission electron microscopy (TEM) and atomic force microscopy (AFM) confirmed multilayer GO and best sheet dispersion at 1 phr, whereas 2 phr showed initial aggregation. Fourier-transform infrared spectroscopy (FTIR) confirmed that the NBR backbone and nitrile groups remained intact, while weak GO-derived C–O–C/C–O bands appeared at higher loadings. The C≡N band at ∼2,237 cm^−1^ preserved its position but showed a slight increase in bandwidth, consistent with the formation of a hydrogen-bonded interphase. X-ray diffraction (XRD) showed loss of GO periodicity in the rubber matrix. UV-Vis/Tauc analysis indicated a non-monotonic band gap (direct 3.01→3.13→3.11 eV; indirect 2.84→2.92→2.96 eV), arising from confinement at well-dispersed loadings and π–π stacking at higher loadings. Dielectric measurements (10^2^–10^6^ Hz, 20 °C–100 °C) evidenced a more stable ε′ for GO-filled samples, maximized at 1 phr. Mechanical testing showed simultaneous gains in tensile strength, tear resistance, and rebound elasticity at low GO loadings, while swelling and thermo-oxidative retention improved due to barrier effects and chain immobilization. Overall, ∼1 phr GO delivers the best structure–property balance, combining hydrogen-bond-mediated interfacial adhesion and optimal dispersion with stable dielectric behavior and reduced swelling/aging sensitivity; 2 phr yields the highest tensile value but also results in incipient aggregation and reduced dielectric stability.

## Graphical Abstract

**Figure:**
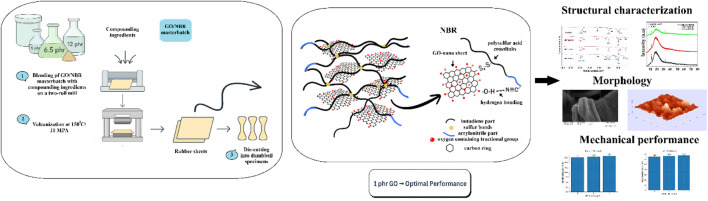

## 1 Introduction

Polymer nanocomposites utilize nanoscale fillers to deliver step-change improvements in mechanical, thermal, and electrical performance, with the *interphase* between filler and matrix widely recognized as the controlling element of property transfer (Mittal et al., 2015; Papageorgiou et al., 2017). Among elastomers, NBR is industrially indispensable for oil- and fuel-exposed components, including seals, gaskets, and hoses, owing to its polarity and chemical resistance; yet, like most non-strain-crystallizing rubbers, unfilled NBR vulcanizates require efficient reinforcement to reach high strength and durability (George et al., 2021). Conventional carbon black achieves this at relatively high phr, but its petroleum origin and processing footprint motivate lower-loading, higher-efficiency alternatives (Mammadov and Khankishiyeva, 2025; Shinde et al., 2022).

GO has emerged as a multifunctional nanofiller that pairs the high aspect ratio and stiffness of the graphene family with abundant oxygenated groups (–OH, –COOH, and epoxide) capable of specific interactions with polar polymers (Chandrasekaran et al., 2013a; Chandrasekaran et al., 2013b; Lim et al., 2021; Mao et al., 2020).

In NBR, these surface functionalities enable hydrogen bonding and strong dipolar coupling to nitrile groups, promoting dispersion, interfacial adhesion, and stress transfer at substantially lower loadings than conventional fillers (Harito et al., 2019). As a result, GO-filled NBR frequently exhibits simultaneous gains in tensile strength, storage modulus, wear resistance, and aging stability at ≤1–2 phr (Mensah et al., 2018a). Beyond mechanics, interfacial polarization and chain immobilization at GO surfaces can also tailor dielectric response and reduce solvent uptake by introducing tortuous diffusion paths and locally increased network density (Badia et al., 2021).

Despite these advances, two gaps persist. First, while good dispersion is often referred to qualitatively, the chemistry of the interphase, particularly the role of hydrogen-bond networks between GO and NBR, and its spectral signature, is less frequently placed at the center of explanation (Valentini et al., 2018; Mensah et al., 2018b). Second, systematic correlations that tie interfacial bonding patterns (as probed by FTIR) to bulk responses spanning mechanics, swelling/crosslink density, and dielectric behavior across a controlled, low GO-loading window remain comparatively limited (Dul et al., 2019). Addressing these gaps is practically important: in nanofiller-rich rubbers, the optimum is rarely at the highest loading. Beyond a critical level, agglomeration and π–π stacking can erode property gains and increase losses (Cai et al., 2020; Mensah et al., 2019).

In this study, we demonstrate that the optimum performance of NBR/GO nanocomposites at low filler content is governed by a hydrogen-bonded interphase. Through controlled GO loadings (0.5–2.0 phr), we identify FTIR spectral markers indicative of hydrogen bonding and dipolar coupling between GO and NBR—namely, the preserved position but slightly increased bandwidth of the nitrile (C≡N) band and the increased intensity of GO-derived C–O–C and C–O features. We correlate these interfacial phenomena with macroscopic property changes, including enhanced tensile and tear strength, rebound elasticity, dielectric behavior across temperature and frequency, and crosslink density inferred from solvent swelling. X-ray diffraction (XRD) and AFM analyses confirm the well-dispersed nature of GO at 1 phr and aggregation at higher loadings, supporting a structure–property relationship in which ∼1 phr GO yields the maximum performance.

Despite the slightly higher tensile strength at 2 phr, AFM/XRD and dielectric analyses indicate that 1 phr is the global optimum, where dispersion is maximized, and interfacial polarization is most stable, consistent with a hydrogen-bonded interphase rather than filler–filler networking. The motivation for the presented study is a detailed evaluation of the structure and optical, morphological, thermal, mechanical, and dielectric properties, as well as an interphase-centered approach that connects hydrogen-bond formation between GO and NBR with macroscopic property optimization at low-filler loadings, which has not been comprehensively addressed in the literature. These findings establish an interphase-centric design principle for achieving high-performance, low-loading nanofiller reinforcement in polar elastomers, with direct application to durable, oil-resistant, and dielectric-stable NBR-based components.

## 2 Experimental

### 2.1 Materials

NBR, with 40 phr acrylonitrile and Mooney viscosity (ML (1 + 4)) of 78 at 100 °C, was supplied by Kocaeli Kauchuk Sanayi A.Ş. (Turkiye). Graphite powder (≥99.9%) for GO synthesis, zinc oxide (ZnO), stearic acid, sulfur, and N-cyclohexyl-2-benzothiazolesulfenamide (CBS) were of analytical grade (Sigma-Aldrich) and used as received. Toluene and other solvents were analytical grade and used without further purification.

### 2.2 Synthesis of GO

GO was prepared via a modified Hummers’ protocol (Guerrero-Contreras and Caballero-Briones, 2015; Sujiono et al., 2020). In brief, 3.0 g of graphite and 1.5 g NaNO_3_ were mixed, followed by the slow addition of 70 mL concentrated H_2_SO_4_ under stirring in an ice bath (0 °C, 1 h). KMnO_4_ (9.0 g) was then added portionwise while keeping the temperature <20 °C; the suspension was maintained for 3 h at 0 °C and subsequently stirred at 35 °C for 1 h. Distilled water (150 mL) was added dropwise (exothermic; temperature rose to ∼98 °C) and held for 30 min, followed by an additional 300 mL of water. Residual Mn species were reduced with 15 mL of 30% H_2_O_2_ until the mixture turned from brown to yellow. The solid was filtered, washed with 1:10 HCl:water and then with water to neutral pH, dried at room temperature, redispersed by ultrasonication (to delaminate), and centrifuged to obtain a stable GO dispersion.

### 2.3 Preparation of GO/NBR masterbatch (solution–coagulation route)

**FIGURE 1:**
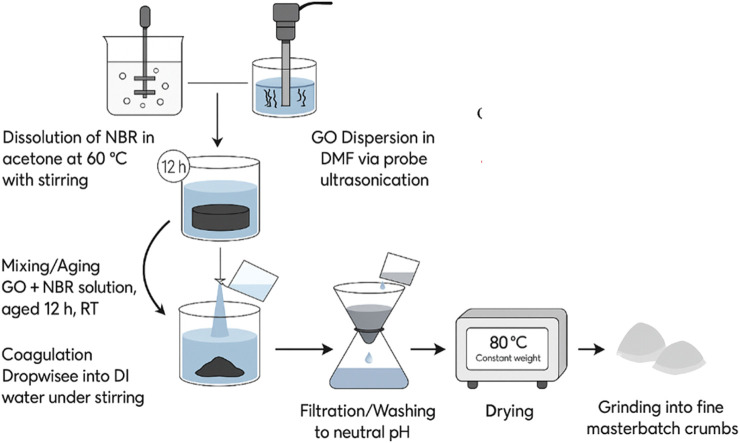
Schematic illustration of the preparation steps for GO/NBR masterbatch via the solution–coagulation method.

NBR was cut into small pieces and dissolved in acetone (400 mL) under vigorous stirring at 60 °C to obtain a homogeneous solution. In parallel, the target amount of GO was dispersed in N, N-dimethylformamide (DMF) by ultrasonication to promote exfoliation and uniform dispersion. The GO/DMF suspension was added dropwise into the NBR/acetone solution under continuous stirring and aged for 12 h to enhance polymer–filler interactions. The composite solution was then coagulated by adding deionized water, and the precipitated GO/NBR masterbatch was filtered and washed thoroughly with water to remove residual solvents/salts. The wet cake was dried in a vacuum oven at 80 °C to constant mass. The process is summarized schematically in Figure 1.

### 2.4 Compounding and vulcanization

**FIGURE 2:**
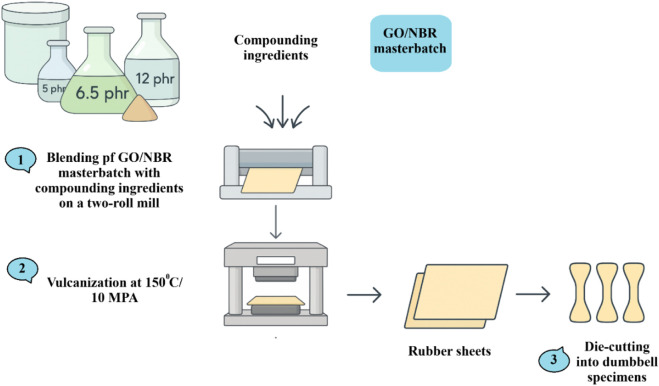
Processing steps for GO/NBR composites.

The dried GO/NBR masterbatch was compounded with pristine NBR (when necessary) and standard vulcanization additives using a laboratory two-roll mill at room temperature. The formulation was homogenized to ensure uniform filler dispersion. The vulcanization system consisted of ZnO, stearic acid, sulfur, and CBS, applied at conventional dosages. The detailed formulations of all GO/NBR compounds are summarized in Table 1. Following mixing, the rubber sheets were conditioned for 24 h at ambient conditions. Cure characteristics were then evaluated using a moving-die rheometer (MDR) in accordance with ASTM D2084, at 150 °C. The processing sequence for GO/NBR composites is presented in Figure 2.

**TABLE 1:** Formulations of GO/NBR compounds (phr).

Sample	NBR (phr)	GO (phr)	ZnO (phr)	Stearic acid (phr)	Sulfur (phr)	CBS (phr)
NBR-0	100	0.00	5.0	1.0	1.5	1.0
NBR–GO0.50	100	0.50	5.0	1.0	1.5	1.0
NBR–GO1.00	100	1.00	5.0	1.0	1.5	1.0
NBR–GO2.00	100	2.00	5.0	1.0	1.5	1.0

Based on the optimum cure time (t_90_) obtained from rheometric curves, the sheets were subsequently press-cured using a hydraulic hot press at 150 °C and 10 MPa. All cured samples were conditioned at room temperature for an additional 24 h before mechanical, thermal, and dielectric testing. The formulations of the GO/NBR compounds are presented in Table 2.

**TABLE 2:** Direct and indirect band gap of different phr GO/NBR composite materials.

E_g_. eV	0.5 phr GO/NBR	1 phr GO/NBR	2 phr GO/NBR
Direct	3.01 eV	3.13 eV	3.11 eV
Indirect	2.84 eV	2.92 eV	2.96 eV

### 2.5 Characterization methods

#### 2.5.1 FTIR spectroscopy

FTIR spectra were collected over 4,000–600 cm^−1^ with a spectral resolution of 4 cm^−1^ and 32 co-added scans per spectrum. Background (air) spectra were recorded before each acquisition and subtracted automatically. Cured rubber sheets (∼1–2 mm thickness) were used directly after the surfaces were cleaned with ethanol and air-dried. All spectra were baseline-corrected and intensity-normalized to the –CH_2_ stretching envelope (2,920–2,850 cm^−1^) to enable semi-quantitative comparison across formulations. Peak positions and full width at half-maximum (FWHM) were extracted by constrained Gaussian fits to track subtle shifts or broadening in the C≡N (∼2,235 cm^−1^), C=C (∼1,640 cm^−1^), and GO-related C=O/C–O–C/C–O (∼1,720, 1,210, and 1,050 cm^−1^) bands (replicates: n = 3 (spectra) per composition).

#### 2.5.2 Equilibrium swelling and crosslink density

Crosslink density was determined by equilibrium swelling in toluene at 23 °C ± 1 °C. Dumbbell off-cuts (∼0.20–0.30 g) were weighed (m_1_), immersed for 72 h until the mass stabilized, blotted, and re-weighed (m_2_). Swollen specimens were vacuum-dried at 80 °C to constant mass (m_3_). Densities used were ρ_s_ (NBR composite) and ρ_s_ (toluene). The composite rubber mass fraction (φ) and any extractive loss during swelling (α) were included to correct for non-rubber constituents (e.g., filler and curatives) (Mensah et al., 2023).

The polymer volume fraction in swollen gel (v_2_m) was calculated as in Equation 1:

$$v_{2m}=\frac{\;m_{1}\times \varphi \times \frac{1-\alpha }{\rho_{s}}\;}{m_{1}\times \frac{1-\alpha }{\rho_{s}}+\frac{m_{2}-m_{3}}{\rho_{s}}\;}.$$ (1)

The elastically effective network chain density υ_e_ (total crosslink density) was obtained from the Flory–Rehner relation, which is presented as Equation 2 (Brandrup et al., 1999; Flory and Rehner, 1943; Rahimova et al., 2022; Paul, 1953).

$$v_{e}=-\frac{\ln(1-v_{2m\;}){+v}_{s2m}+\chi v_{2m}^{2}}{v_{s}\left(v_{2m}^{\frac{1}{3}}-\frac{v_{2m}}{2}\right)},$$ (2)

where Vs is the molar volume of toluene (107 cm^3^ mol^−1^) and χ is the Flory–Huggins interaction parameter for NBR (≈40% ACN)/toluene (taken from literature at the measurement temperature). Each value is reported as mean ± standard deviation (SD (n = 3)).

#### 2.5.3 Mechanical properties testing

Mechanical performance was evaluated by tensile, tear, hardness, rebound, adhesion, and thermal aging tests. Tensile strength, elongation at break, and modulus were measured using a ZwickRoell Z020 universal testing machine (ISO 37:2017) at a crosshead speed of 500 mm/min. Tear resistance was evaluated according to ISO 34-1:2015, Method B. Hardness (Shore A) was assessed using a TM-2 durometer (ISO 7619-1:2010). Rebound elasticity was tested in accordance with ISO 4662:2017. Adhesion to metal was determined using ASTM D429 Method B. Thermal aging behavior was assessed by comparing tensile and elongation values before and after oven treatment at 397 K for 48 h (ISO 188:2011).

#### 2.5.4 Structural and morphological characterization

XRD analysis was performed using a Rigaku MiniFlex 600 diffractometer with Ni-filtered Cu Kα radiation (λ = 1.5406 Å) in the 2θ range of 0°–80° to study GO structure and interlayer spacing changes after composite formation. Scanning electron microscopy (SEM) and energy-dispersive X-ray spectroscopy (EDS) were carried out using a JSM-6490LV JEOL and a VEGA Tescan SEM at 20.0 kV to examine dispersion, morphology, and element analysis. TEM with JEOL JEM-1400 at 80–120 kV enabled nanoscale observation of GO sheets and their alignment within the rubber matrix. Absorbance spectra were determined using UV-visible (UV-Vis) spectroscopy (Specord 250 Plus) in the 190–1,100 nm range. This provided insights into π–π* transitions, conjugation effects, and potential electronic modifications introduced by GO in the elastomer matrix. Dielectric properties were assessed using the E7-20 instrument (MNIPI, Belarus) under varying electric field conditions. This analysis helped characterize charge transport and interfacial polarization effects introduced by GO, which are critical for applications in sensing and electromagnetic interference (EMI) shielding.

## 3 Results and discussion

### 3.1 FTIR analysis

**FIGURE 3:**
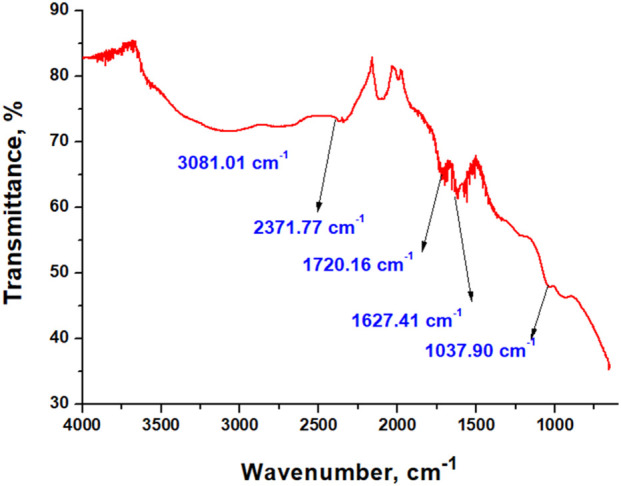
FTIR spectra of pure GO.

Chemical bonds between elements can be identified by analyzing the infrared transmittance spectra of materials. The results obtained from the study of the FTIR spectra of pure GO are presented in Figure 3.

The recorded spectrum of GO displays characteristic absorption bands at 3,081.01 cm^−1^ (broad O–H/C–H stretching), 2,371.77 cm^−1^ (adsorbed CO_2_), 1,720.16 cm^−1^ (C=O stretching of carboxylic/edge carbonyls), 1,627.41 cm^−1^ (C=C skeletal vibrations or adsorbed water), and 1,037.90 cm^−1^ (C–O stretching), confirming the presence of oxygenated functional groups consistent with literature reports (Guerrero-Contreras and Caballero-Briones, 2015; Sujiono et al., 2020).

**FIGURE 4:**
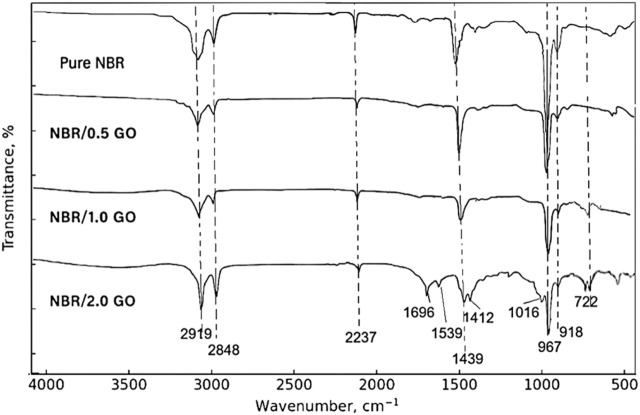
FTIR spectra of pure NBR and NBR/GO nanocomposites (0.5 phr, 1.0 phr, and 2.0 phr GO).

Figure 4 presents the spectra of neat NBR and NBR/GO nanocomposites containing 0.5 phr, 1.0 phr, and 2.0 phr GO.

For a meaningful comparison, the spectra were baseline-corrected and normalized to the intensity of the CH_2_ stretching band at 2,919 cm^−1^. Pure NBR exhibits the characteristic CH_2_ stretching doublet at 2,919 cm^−1^ and 2,848 cm^−1^, a distinct nitrile stretching band at 2,237 cm^−1^, CH_2_ deformation bands at 1,439 cm^−1^ and 1,361 cm^−1^, and microstructural markers at 967 cm^−1^, 918 cm^−1^, and 668 cm^−1^ assigned to trans-1,4-, 1,2-vinyl and cis-1,4-butadiene units, respectively, in agreement with reported spectra for NBR (Aliyeva et al., 2023; Harito et al., 2019).

After incorporation of 0.5 phr and 1.0 phr GO, all main NBR bands are retained and appear at essentially the same wavenumbers. The ν(C≡N) band remains centered at approximately 2,237 cm^−1^ for all compositions and shows no measurable frequency shift within the experimental resolution. The most noticeable changes are a slight broadening of the 2,237 cm^−1^ band and a small increase in the width of the CH_2_ deformation region, where the 1,439 cm^−1^ band evolves into a somewhat broader feature at approximately 1,435–1,439 cm^−1^. The microstructural bands at 967 cm^−1^, 918 cm^−1^, and 667–668 cm^−1^ remain well resolved, indicating that the butadiene sequence distribution in the NBR backbone is not significantly affected by these filler levels (Mammadov et al., 2025; Mensah et al., 2018a).

At 2.0 phr GO, additional weak bands appear in the fingerprint region. A shoulder near 1,696 cm^−1^ and bands at 1,539 cm^−1^ and 1,412 cm^−1^ can be attributed to the C=O and C=C–O vibrations of GO-derived oxygenated moieties, while new features at 1,212 cm^−1^ and 1,016 cm^−1^ are assigned to epoxy C–O–C and alkoxy C–O groups. Peaks at 722 cm^−1^ and 668 cm^−1^ are associated with out-of-plane vibrations of substituted olefinic and methylene groups. The NBR-specific CH_2_, C≡N, and butadiene bands are clearly present, confirming that the polymer backbone remains intact during composite preparation. The trans-1,4-butadiene band at 967 cm^−1^ becomes slightly broader and less intense at 2 phr GO, which is consistent with somewhat restricted chain mobility in GO-rich interphases.

Overall, the FTIR data show that GO is successfully introduced into the NBR matrix without generating new covalent-bond signatures. The preserved position of the nitrile band, together with its modest broadening and the emergence of GO-related C=O and C–O bands, points to an interface dominated by physical interactions, primarily hydrogen bonding between NBR nitrile groups and GO hydroxyl/carboxyl functionalities, assisted by dipole–dipole interactions between polar groups, rather than extensive chemical grafting (Mammadov, 2024). These non-covalent interactions provide a reasonable microscopic basis for the improved dispersion and interfacial adhesion, and for the property enhancement observed particularly at 1.0 phr GO.

**SCHEME 1:**
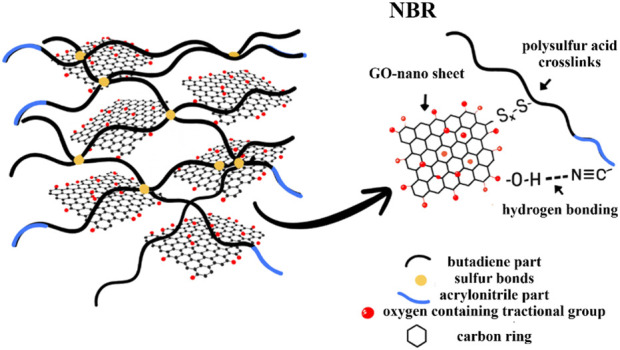
Schematic of the hydrogen-bonded interphase in NBR/GO nanocomposites.

As illustrated in Scheme 1, nitrile–GO hydrogen bonding forms a polar interphase that rationalizes the spectral changes and the property optimum at ∼1 phr GO. Polar C≡N groups on NBR form H-bonds (C≡N···H–O) with oxygenated groups and band broadening on GO nanosheets.

These non-covalent interactions play a crucial role in interfacial adhesion and filler dispersion. The progressive spectral evolution supports the hypothesis that GO introduces a localized polar environment, altering the dipole moment distributions in NBR, especially near the nitrile and butadiene sites. This behavior aligns with previous reports that demonstrate enhanced mechanical properties and thermal stability due to the formation of hydrogen-bonding networks in similar rubber nanocomposite systems (Zhang et al., 2022).

Overall, FTIR spectroscopy confirms that while the chemical identity of the NBR matrix is preserved, the incorporation of GO alters the molecular environment via physical interactions. These interactions, particularly hydrogen bonding, promote interfacial compatibility and are foundational to the composite’s performance enhancements.

### 3.2 Structural analysis

**FIGURE 5:**
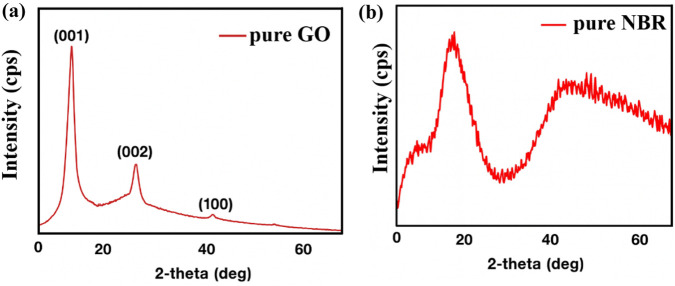
XRD patterns of pure GO **(a)** and NBR **(b)**.

XRD analysis is essential to determine the structure of nanocomposite materials, as well as to calculate crystallite size. Pure GO (a) and NBR (b), which were used as the initial matrices, were analyzed by XRD analysis. The diffractogram is shown in Figure 5.

**FIGURE 6:**
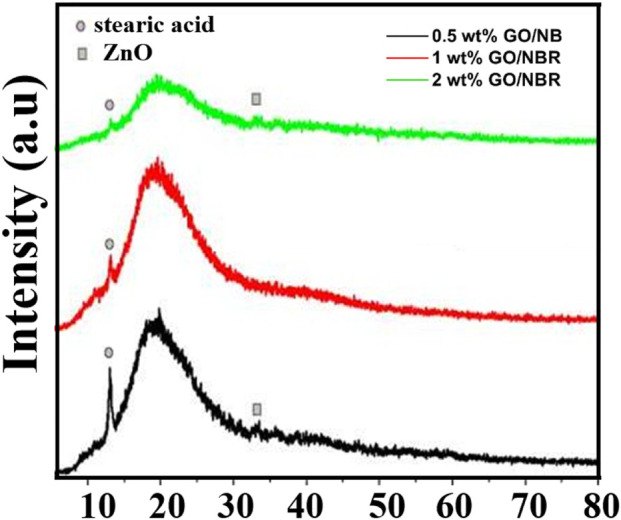
XRD patterns of different percentages (0.5 phr, 1 phr, and 2 phr) of GO/NBR.

In the diffraction pattern of pure GO (Figure 5a), peaks were observed at angles 2θ = 11.98°, 26.30°, 42.04°, and 54.39°. These peaks are denoted by the Miller indices (001), (002), and (100), respectively (Gahramanli et al., 2023). In the diffraction pattern of pure NBR (Figure 5b), a broad characteristic bulge at 2θ = 20° interval is observed, which is related to the amorphous structure of NBR. There is no crystalline structure here. A comparison with the literature confirms that the observed diffraction pattern belongs to pure NBR (Ateia et al., 2023; Kapgate et al., 2012; Thomas et al., 2011). The XRD patterns of different concentrated GO/NBR nanocomposite materials are given in Figure 6.

In the 0.5 phr GO/NBR composite, XRD reveals peaks at 2θ = 12.98° and 33.27°. In the scientific literature, 2θ ∼ 12°,16°, and 24° correspond to pure stearic acid diffraction peaks. In the 0.5 phr GO/NBR composite, XRD reveals a peak at 2θ ≈ 12.98°, which corresponds to stearic acid, utilized as an initial agent during the composite preparation stage (Chaichan et al., 2017; Habib et al., 2017). This stearic acid-related reflection is still visible for the 1 phr GO/NBR sample, although with lower intensity. However, this peak significantly decreased in the 2 phr GO/NBR composite. Because the amount of stearic acid is the same in all composites, the significant decrease in this peak at 2 phr GO is attributed to a change in its physical state. At higher GO loading (2 phr GO/NBR), stearic acid is strongly adsorbed on the GO surface, so the intensity of this peak decreased. Overall, the XRD patterns align well with those reported in the literature (Habib et al., 2017; Yong and Munusamy, 2023).

Thus, the main diffraction pattern is observed at angles as wide as ∼20°, as in NBR. The characteristic peak of NBR in the diffraction pattern is observed more intensely, and the characteristic peak of GO is not observed sharply due to its small amount. In the literature, it is reported that this indicates that the periodic crystalline structure of GO vanished in the rubber in the GO/NBR composite materials, and GO was fully exfoliated into a monolayer or a few layers in the NBR matrix (Heinrich, 2011), where the exfoliation depends on the processing technique and the affinity between the sheets. Thus, as the concentration of GO increases, the percolation load of GO in NBR shows up via the reduction of broad peak intensity at 2 phr GO/NBR.

The diffraction peaks between 30° and 35° are assigned to ZnO particles in the vulcanizates (El Mejjatti et al., 2014; Premanathan et al., 2011). The small intensity peak (bump) observed at angles of approximately ∼33° for these composites is attributed to ZnO. In the literature, the diffraction peak observed at this angle (002) has been further confirmed to be attributed to ZnO by being denoted by the Miller index (Kataru et al., 2018). All the samples show no obvious characteristic peaks of graphite or GO, indicating that GO sheets are homogeneously dispersed in the polymer matrix and do not show a graphite-like ordered structure.

In the XRD analysis, the intensity in the range 2θ = 12.98°–13.08° follows the order 0.5 phr GO/NBR >1 phr GO/NBR >2 phr GO/NBR. As the GO content in the NBR matrix increases, GO becomes more densely distributed, and the intensity of this diffraction peak decreases markedly. In the 2-phr GO/NBR composite, the already weak stearic acid signal is almost completely masked by the broad diffraction background of the GO/NBR matrix, which explains the pronounced reduction in intensity near 2θ ≈ 13°. At the same time, the intensity in the interval 2θ = 18°–20° is as follows: 1 phr GO/NBR >0.5 phr GO/NBR >2 phr GO/NBR. Here, the change in intensity is related to the orderly arrangement of the polymer chains. Thus, because the amount of GO in the 0.5-phr GO/NBR composite is small, its interaction with the polymer chains is weak, and little ordering of the polymer chains occurs. In the 1-phr GO/NBR composite, GO structures are well distributed in the composite and can interact better with the polymer chains, leading to a more regular arrangement of the polymer chains. Therefore, the intensity is maximal here. Because the amount of GO structures in the 2-phr GO/NBR composite is higher, it accumulates and aggregates, and as a result, the chains are arranged irregularly, causing a decrease in intensity.

### 3.3 Microscopy and element analysis

**FIGURE 7:**
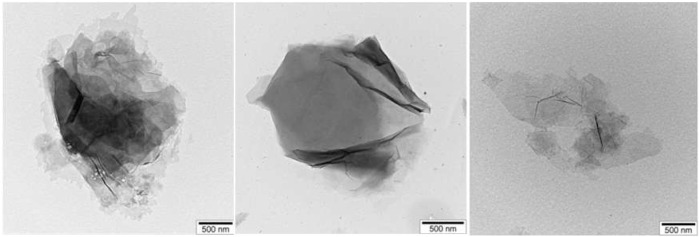
TEM images of pure GO.

The morphology and elemental composition of the samples were analyzed using SEM, TEM, and EDS techniques. The TEM results of pure GO are presented in Figure 7.

**FIGURE 8:**
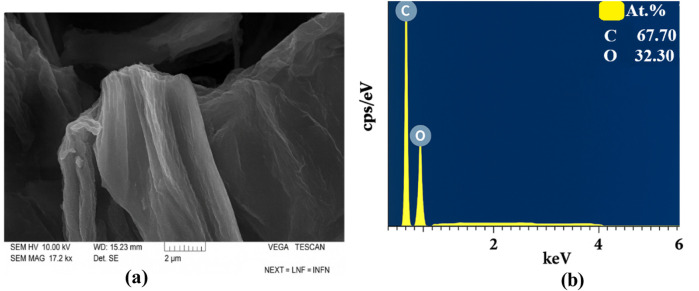
**(a)** SEM images and **(b)** EDS results of pure GO.

TEM shows few-layer GO sheets. SEM images of GO also showed that they formed in a layered form, and element analysis revealed no additional residual elements. The SEM image of GO and the results of element analysis are shown in Figure 8.

Element analysis determined that C is 67.7% and O is 32.3%. There are no residual elements.

**FIGURE 9:**
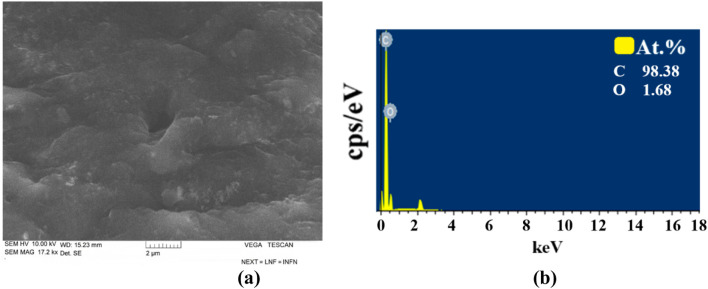
**(a)** SEM images and **(b)** EDS results of pure NBR.

Figure 9 shows the SEM images (Figure 9a) and EDS (Figure 9b) results of pure NBR.

Based on the results of SEM, NBR is composed of both small and large microcracks. At the same time, from EDS analysis, it was found that it contains 98.38% C and 1.68% S elements.

**FIGURE 10:**
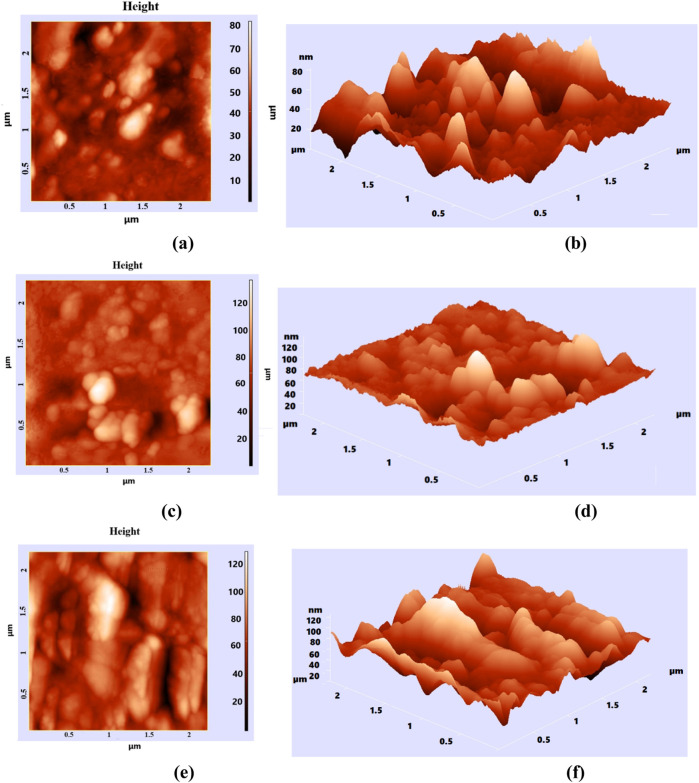
AFM images of 0.5 phr GO/NBR (2D **(a)**, 3D **(b)**); 1 phr GO/NBR (2D **(c)**, 3D **(d)**); and 2 phr GO/NBR (2D **(e)**, 3D **(f)**).

AFM is an essential technique for obtaining detailed information about the sample surface, including its topography, two-dimensional (2D) and three-dimensional (3D) images, and data on the arrangement density and histograms of nanostructures in the nanometer range. Figure 10 illustrates the 2D (Figures 10a,c,e) and 3D (Figures 10b,d,f) images of the composition and surface distribution of different phr GO (0.5 phr, 1 phr, and 2 phr) in NBR.

The surface of pure NBR is a rough material. Here, different phr of GO are reinforced inside NBR and variously distributed within NBR. The introduced GO is located not only on the surface of NBR but also inside it as a result of vulcanization. From the AFM images, with the increase in the phr of GO in NBR, the coating of the NBR surface by GO leads to a decrease in roughness. This is seen from the 3D images of 1 phr and 2 phr GO/NBR composite materials. Here, at 0.5 phr, due to the low amount of fillers, the cracks inside and on the surface of NBR are not completely filled, and there is a lot of roughness, while at 1 phr, many cracks are filled with filler, and it is considered the most optimal concentration at which GO is dispersed well in the NBR matrix. At 2 phr, the amount of GO is high, and the phenomenon of aggregation–agglomeration occurs in the cracks, which is clearly visible from the 3D image (Figure 10f).

**FIGURE 11:**
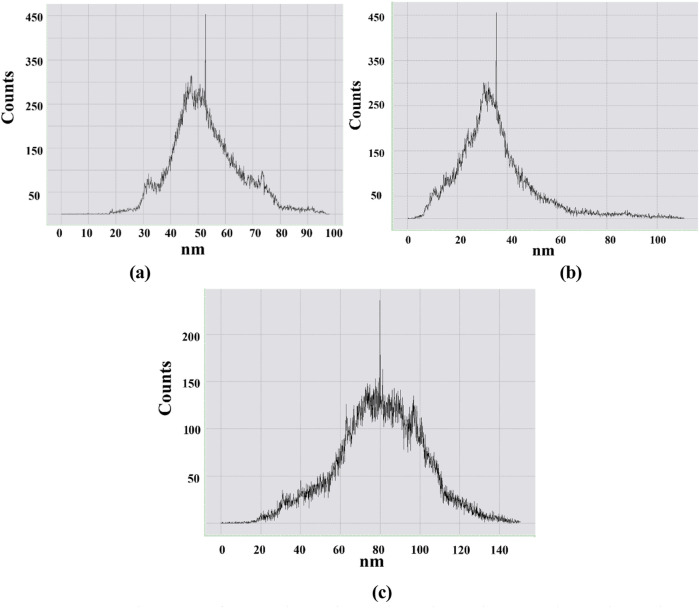
Histogram of **(a)** 0.5 phr GO/NBR, **(b)** 1 phr GO/NBR, and **(c)** 2 phr GO/NBR.

At the same time, histograms were also presented to investigate the size distribution density of nanostructures formed as a result of GO distribution in the mentioned image region in Figure 11.

According to the presented histograms, the range in which nanostructures were formed at 0.5 phr GO was 40–60 nm; at 1 phr, it was mainly 20–45 nm, and at 2 phr, it was 60–100 nm. Nanoparticle sizes obtained from the histogram correlate with XRD results. At 0.5 phr, GO is not fully dispersed within NBR but is locally aggregated, and this interval is related to incomplete dispersion. Therefore, in the XRD pattern of 1 phr GO/NBR composite material, GO particles are well distributed in the NBR and can interact better with the polymer chains, leading to a more regular arrangement of the polymer chains. Therefore, the intensity is maximal here. This can be clearly seen from the 3D image in the AFM images (Figure 10d). The next result from XRD, that agglomeration occurs with increasing GO content, is further confirmed by the AFM images, where the roughness of the NBR is reduced.

### 3.4 UV-Vis spectroscopy

**FIGURE 12:**
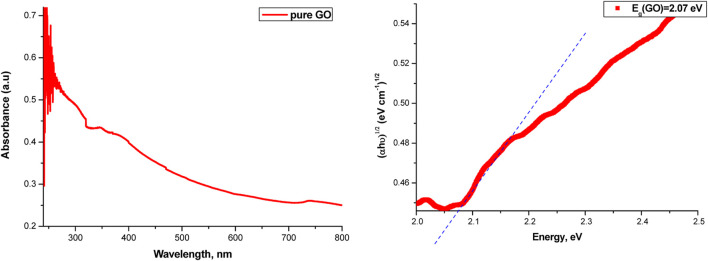
Absorbance spectrum (left) and band gap value (right) of pure GO.

The absorption curves of the samples in the range of 200–800 nm, as well as the determination of the band gap value for the samples from these curves, are possible using the Tauc relation. The absorption curves for GO and the determination of the band gap value are given in Figure 12.

**FIGURE 13:**
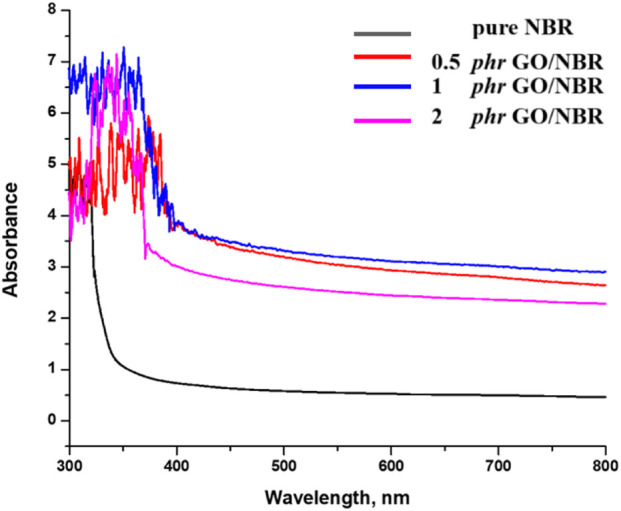
Absorbance spectrum of different percentages of GO/NBR composite materials.

The absorbance spectrum for pure NBR and the absorbance spectra of the composite materials are given in Figure 13.

**FIGURE 14:**
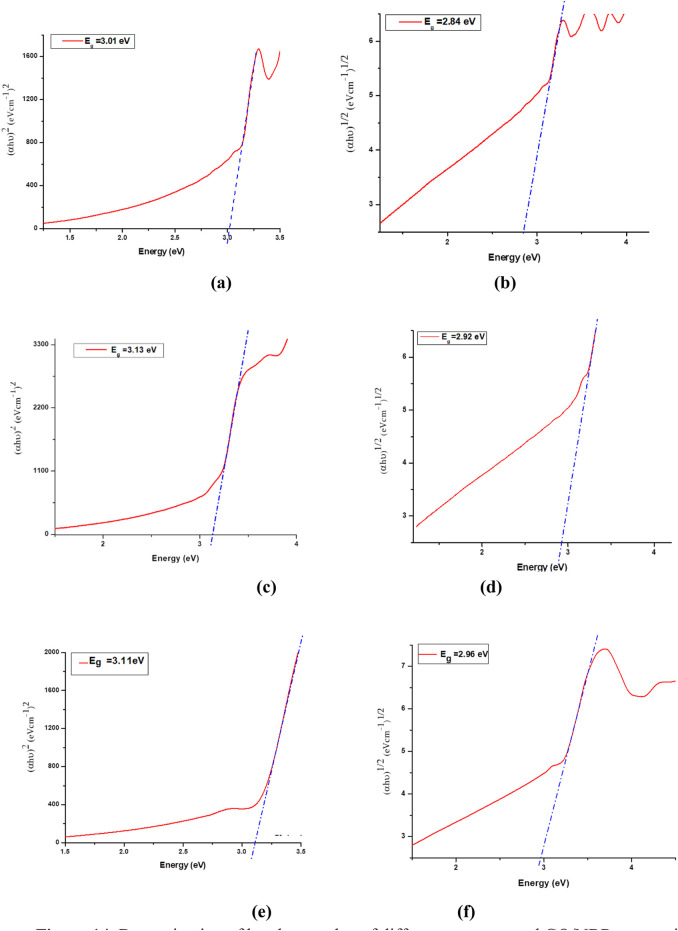
Determination of band gap value of different concentrated GO/NBR composite materials by the Tauc relation. Direct band gap: **(a)** 0.5 phr GO/NBR, **(b)** 0.5 phr GO/NBR, **(c)** 1 phr GO/NBR, **(d)** 1 phr GO/NBR, **(e)** 2 phr GO/NBR indirect band gap, and **(f)** 2 phr GO/NBR.

The band gap values of the composite materials were determined using Tauc plots from the obtained absorbance spectra and are presented in Figure 14.

The direct and indirect band gap of different phr levels of GO reinforced in NBR polymer matrix are presented in Table 2.

The incorporation of GO into an NBR matrix at concentrations ranging from 0.5 phr to 2 phr results in a non-monotonic variation in both direct and indirect optical band gap values. Specifically, the optical band gap initially increases as the GO content rises from 0.5 phr to 1 phr, with the indirect band gap increasing from 2.84 eV to 2.92 eV and the direct band gap increasing from 3.01 eV to 3.13 eV. Upon further increasing the phr GO content, the indirect band gap continues to increase slightly to 2.96 eV, while the direct band gap decreases slightly to 3.11 eV.

This complex behavior is primarily attributed to the dispersion and aggregation of GO within the polymer matrix and interfacial electronic hybridization between GO sheets and NBR chains. At lower GO concentrations (0.5–1 phr), GO is better dispersed within the NBR matrix than at higher concentrations (2 phr), facilitating uniform filler distribution and enhancing interfacial interactions. These well-dispersed nanodomains restrict the movement of charge carriers, inducing quantum confinement effects. According to the “particle-in-a-box” model, this spatial restriction raises the energy levels of confined electrons, resulting in a blue shift—an increase in band gap energy (de Lima et al., 2020; Qadoos et al., 2025).

In addition, uniform dispersion passivates localized trap states, improves polymer–filler interfacial coupling, and increases the effective conjugation length, collectively contributing to the observed band gap enlargement. However, at higher GO loadings (approximately 2 phr), aggregation becomes more pronounced. The increased π–π stacking between GO sheets leads to the formation of extended conjugated domains and partial restoration of the sp^2^ carbon network (de Lima et al., 2020; Qadoos et al., 2025; Zainab et al., 2023). This structural reorganization increases charge carrier delocalization, reduces the spatial confinement effect, and diminishes interfacial hybridization. As a result, a slight reduction in the direct band gap (to 3.11 eV) is observed, consistent with the redshift in the UV-Vis absorption spectra. Nevertheless, the indirect band gap continues to increase slightly, possibly due to persistent localized interfacial states and residual confinement effects that remain active despite aggregation. The UV-Vis absorption spectroscopy further supports these findings. Pure NBR exhibits a wide absorption edge in the UV region. The incorporation of GO results in a progressive redshift in the absorption edge, indicating increased light absorption at longer wavelengths. This shift reflects the narrowing of the optical band gap and corresponds to enhanced π–π* transitions, stronger electronic coupling, and structural modifications within the composite. Notably, the emergence of this redshift at only 0.5 phr GO suggests the early formation of hybrid states through interfacial charge transfer between GO and the NBR matrix. The effect becomes more pronounced at 1 phr GO loading, and although partial aggregation at 2 phr reduces some interfacial effects, the overall redshift trend persists.

The Tauc plot analysis (Figure 14) further confirms this behavior, showing a systematic decline in the optical band gaps, especially at lower concentrations. This decline aligns with the partial restoration of π-electron delocalization as GO content increases and oxygenated functional groups are gradually reduced (de Lima et al., 2020; Qadoos et al., 2025; Zainab et al., 2023).

Despite a general expectation of a decreasing band gap with increasing GO content due to π–π stacking and sp^2^ restoration, the experimental data reveal a temporary band gap increase at 1 phr GO. This deviation is explained by well-dispersed GO at this concentration, leading to a higher degree of electronic localization and interfacial passivation.

### 3.5 Dielectric measurements

**FIGURE 15:**
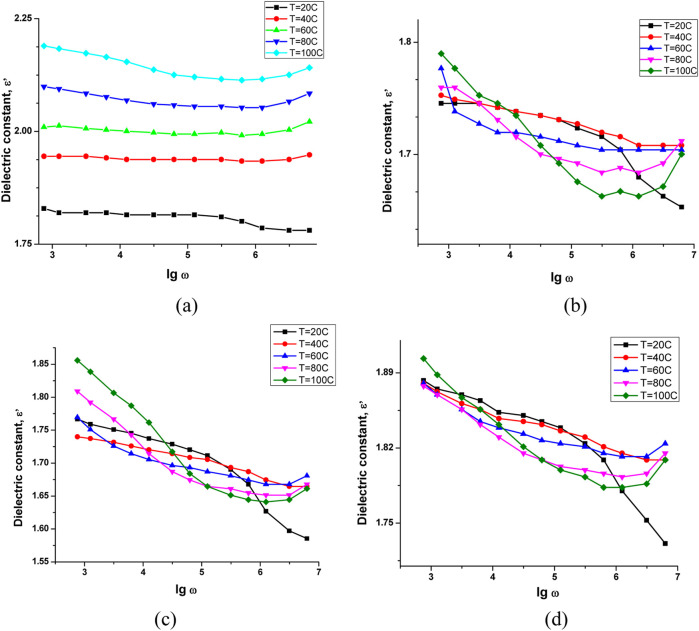
Frequency dependence of the dielectric constant of **(a)** pure NBR, **(b)** 0.5 phr GO/NBR, **(c)** 1 phr GO/NBR, and **(d)** 2 phr GO/NBR.

The frequency dependence of the dielectric constant of nanocomposite materials was studied at different temperatures (20 °C–100 °C), and the results are presented in Figure 15.

The dielectric constant (ε′) of pure NBR increases with temperature and decreases with frequency. At T = 20 °C, ε′ = 1.8289 at low frequency (lgω = 2.87), decreasing with frequency. As the temperature rises, ε′ at low frequency increases to 1.9446 (40 °C), 2.0096 (60 °C), 2.0992 (80 °C), and 2.1897 (100 °C). At high frequencies (lgω ≈6.79), a slight increase in ε′ is observed at higher temperatures, with values like 2.0215 (60 °C) and 2.1412 (100 °C), indicating increased dipole mobility and thermally activated charge transport. The behavior reflects that at low frequencies, dipoles respond well to the electric field, but at higher frequencies, they delay, leading to a drop in ε′. However, increased thermal energy enhances their reorientation ability and interfacial polarization, thus boosting ε′ (Kumar et al., 2015; Thomas and Manoj, 2021).

For 0.5 phr GO/NBR, ε′ also increases with temperature at low frequency, from 1.7455 (20 °C) to 1.7898 (100 °C), while decreasing with frequency. At high frequencies (lgω = 6.79), ε′ gradually rises from 1.7041 (60 °C) to 1.7119 (80 °C), followed by a slight decrease at 100 °C. This trend is linked to β-relaxation and Maxwell–Wagner–Sillars (MWS) polarization, which dominates at low frequencies, and tunneling mechanisms that become significant at higher frequencies. At 60 °C, increased interfacial activity leads to a temporary ε′ rise, while at 80 °C, local disorder reduces the dipole response. At 100 °C, ε′ rises again due to reactivation of charge carrier movement (Vennemann et al., 2020).

In 1 phr GO/NBR, a similar trend is observed: ε′ increases from 1.7667 (20 °C) to 1.8558 (100 °C) at low frequency. At high frequencies, ε′ rises from 1.5854 (20 °C) to 1.6807 (60 °C), then slightly drops, showing a peak in interfacial charge transport efficiency at 60 °C. This trend reflects two competing effects: confinement at well-dispersed loadings and π–π stacking when GO aggregates.

For 2 phr GO/NBR, ε′ at low frequency increases slightly from 1.8828 (20 °C) to 1.9033 (100 °C). At high frequency (lgω = 6.79), ε′ increases from 1.7309 to 1.8088 (100 °C), peaking at 1.8241 at 60 °C. Increasing GO concentration increases ε′ at low frequencies due to stronger MWS polarization, more interfacial charge accumulation, and formation of tunneling.

Overall, ε′ decreases with frequency for all samples due to dipole delay time and increases with temperature due to increased dipole alignment and charge mobility. GO addition raises ε′ at low frequencies via MWS polarization and promotes hopping conduction at higher frequencies. However, in composites, the temperature-induced rise in ε′ is less sharp than pure NBR, as GO restricts dipole flexibility and introduces a more stable interfacial structure. At high GO contents, sp^2^-hybridized domains increase tunneling, and the material enters a near- or post-percolation regime, where ε′ becomes less sensitive to temperature changes (Amirli et al., 2025; Xia et al., 2017).

Thus, GO incorporation improves dielectric behavior by stabilizing ε′ across temperature and frequency, with a pronounced increase at low frequencies due to interfacial effects and at high frequencies via hopping and tunneling, while pure NBR shows a sharper ε′ increase with temperature due to unrestricted dipole motion and thermal activation (Badia et al., 2021).

The GO/NBR nanocomposite containing 1 phr GO exhibited the most pronounced and stable dielectric performance across the tested temperature and frequency ranges. This enhancement is primarily attributed to the homogeneous dispersion of GO nanosheets within the NBR matrix, which leads to a well-developed interfacial region between the filler and the polymer chains. At this concentration, the GO sheets are well-separated, minimizing agglomeration and thereby allowing for more effective interfacial polarization (Maxwell–Wagner–Sillars effect). The presence of these interfaces facilitates space-charge accumulation, contributing to a higher dielectric constant at low frequencies without significantly increasing dielectric loss.

Unlike the 0.5 phr composite, which lacks sufficient filler to establish a percolative network, and the 2 phr composite, where partial agglomeration disrupts the homogeneity, the 1 phr composite achieves an optimal balance between filler loading and matrix interaction. As the frequency increases, the dielectric constant of the 1 phr composite shows a gradual and consistent decrease, indicative of a typical dielectric relaxation behavior where dipolar orientation cannot follow the rapid alternation of the applied field. However, the relatively smooth decay compared to other samples indicates suppressed interfacial charge scattering and lower energy loss mechanisms.

Additionally, the dielectric performance of the 1 phr GO/NBR composite was found to improve further with increasing temperature, reflecting enhanced segmental mobility of polymer chains and increased dipolar orientation. Importantly, the composite maintains dielectric stability without the irregular behavior observed in the 2 phr sample, affirming the thermal and electrical compatibility of GO at this loading. These observations strongly support the conclusion that 1 phr GO is the optimum concentration for maximizing dielectric performance in GO/NBR systems through synergistic dispersion, interfacial interaction, and network formation.

### 3.6 Mechanical properties

**FIGURE 16:**
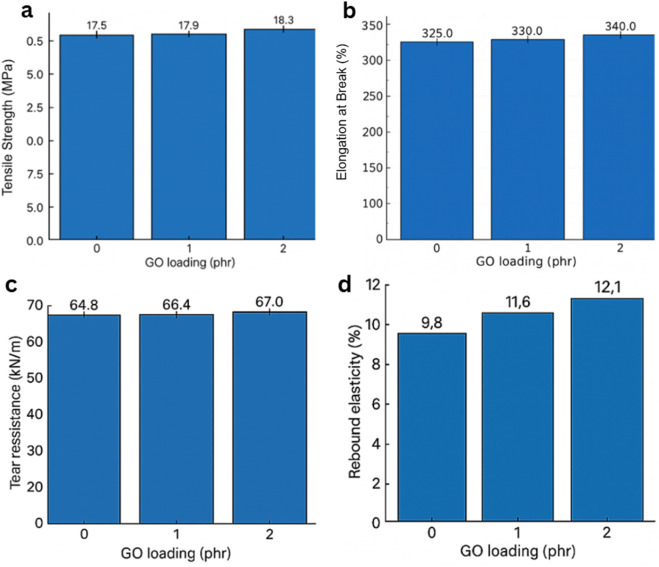
Mechanical performance of GO/NBR nanocomposites as a function of GO loading (0–2 phr). **(a)** Elongation at break, **(b)** tensile strength, **(c)** tear resistance, and **(d)** rebound resilience.

The mechanical performance of the GO/NBR nanocomposites was thoroughly investigated to assess the impact of varying GO concentrations (0.5 phr, 1 phr, and 2 phr) on the structural integrity, toughness, and durability of NBR, and the results are shown in Figure 16. The objective was to optimize the mechanical behavior for engineering applications by using the unique reinforcement capabilities of GO.

Tensile strength increased slightly from 17.5 MPa (pure NBR) to 17.9 MPa at 1 phr and 18.3 MPa at 2 phr (≈5% over pure). Elongation at break rose from ∼325% (pure) to ∼330% (1 phr) and ∼340% (2 phr). These gains are consistent with strong polymer–filler coupling: polar groups on GO (–OH, –COOH, and epoxide) interact with the nitrile-bearing NBR backbone, promoting bound-rubber formation and improved stress transfer across the interface (Mensah et al., 2018a).

Despite the slightly higher tensile at 2 phr, AFM/XRD and dielectric analyses indicate that 1 phr is the global optimum, where dispersion is maximized, and interfacial polarization is most stable, consistent with a hydrogen-bonded interphase rather than filler–filler networking.

GO sheets also act as physical crosslinking constraints within the vulcanized network, which delays strain localization and enables the composite to sustain higher loads before failure. At higher loading, the risk of micro-agglomeration rises; thus, improvements remain moderate rather than dramatic, which is typical for low-phr nanocarbon in elastomers (Figure 16C) (Sr et al., 2018).

Tear strength increased from ∼64.8 kN m^−1^ (pure) to ∼66.4 kN m^−1^ at 1 phr and ∼67.0 kN m^−1^ at 2 phr. The toughening mechanism is classical crack path deflection/bridging by high-aspect-ratio platelets: dispersed GO sheets act as obstacles that force the crack to the nonlinearity branch, or pin, thereby increasing the energy required for propagation (Mensah et al., 2018a; Sr et al., 2018).

**FIGURE 17:**
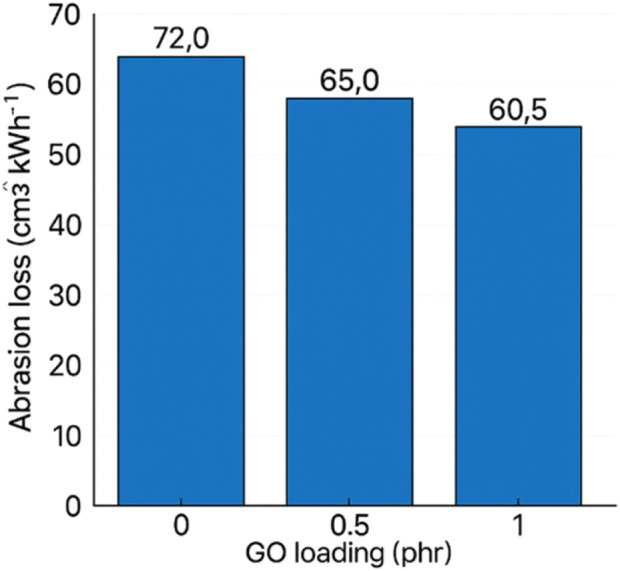
Abrasion loss of GO/NBR as a function of GO loading (0–2 phr) hardness (Shore A).

GO markedly reduced wear; the abrasion loss decreased gradually with loading, reaching ∼58.5 cm^3^ kW h^−1^ at 2 phr. Literature similarly reports that GO can form a thin, load-bearing interphase and hinder material removal under friction, correlating with improved surface integrity in sliding contact (Krishnamurthy and Chandran, 2025; Mensah et al., 2018a). The result is meaningful for seals and dynamic rubber parts where abrasive wear governs service life, as shown in Figure 17.

A slight softening was observed from ∼82 (pure) to ∼77 at 2 phr. Although hard fillers often raise hardness, GO’s interplay with sulfur cure chemistry and local network rearrangement can redistribute crosslinks and slightly increase free volume in confined domains; small agglomerates may also behave as compliant inclusions. The change is limited and does not compromise stiffness in a functionally significant way at these loadings (Sr et al., 2018).

**FIGURE 18:**
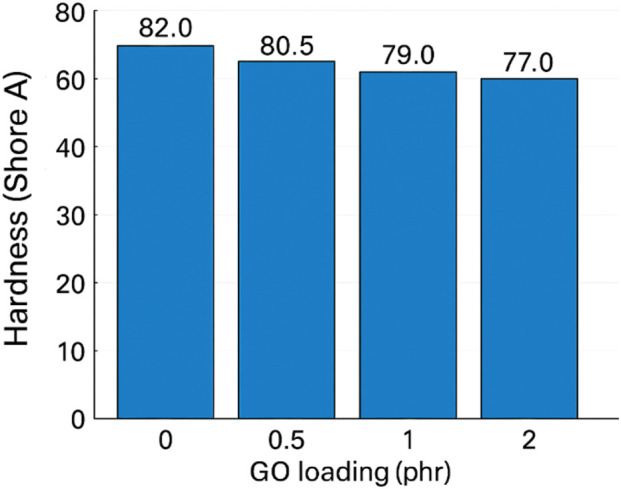
Hardness (Shore A) of GO/NBR as a function of GO loading (0–2 phr).

Hardness of GO/NBR as a function of GO loading is demonstrated in Figure 18.

Within 0.5–2.0 phr, GO consistently improves strength, ductility, tear, rebound, and wear, with the best combined balance typically in the 1–2 phr range. Further increases risk aggregation and diminishing returns, reflecting the known trade-off between polymer–filler and filler–filler interactions in graphene-reinforced elastomers (Mensah et al., 2018b; Sr et al., 2018). These findings support GO as a practical, low-loading reinforcement for engineering-grade NBR components.

**FIGURE 19:**
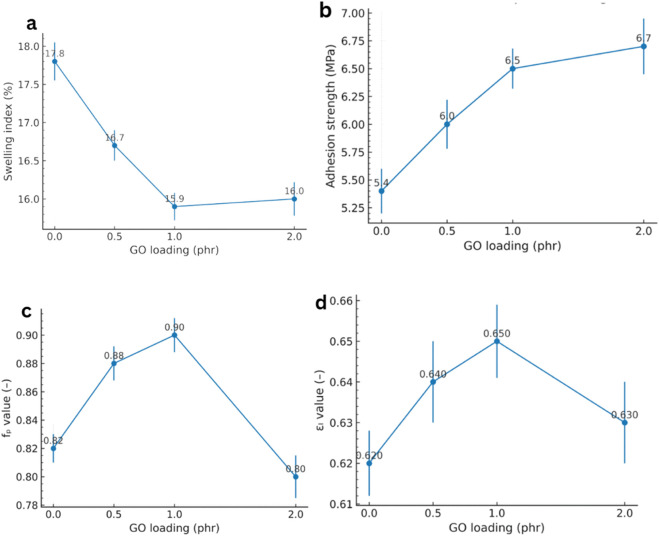
Performance of GO/NBR nanocomposites vs. GO loading (0–2 phr): **(a)** swelling in toluene (24 h, 25 °C), **(b)** rubber–metal pull-out strength (MPa), **(c)** strength retention (f_p_), and **(d)** elongation retention (ε_l_) after aging.

The performance of GO/NBR nanocomposites vs. GO loading (0–2 phr) is shown in Figures 19a–d. GO markedly reduced the solvent swelling of NBR in a benzene/benzole (3:1, v/v) mixture. As shown in Figure 19A, the equilibrium swelling degree exhibits a clear minimum at 1 phr GO and only a slight recovery at 2 phr, consistent with an optimum governed by dispersion.

Quantitatively, swelling drops from ∼17.8% for pure NBR to ∼15.9% at 1 phr, evidencing a substantial improvement in solvent resistance (±SD, n = 3). This trend arises from two complementary mechanisms. (i) An effective rise in crosslink density: oxygenated functionalities on GO act as key points or participate in interfacial reactions with the rubber, lowering the molecular weight between crosslinks and restricting free volume. (ii) A nonlinear diffusion path introduced by plate-like GO, which lengthens solvent trajectories and suppresses permeation—a classical barrier effect of layered nanofillers (Mensah et al., 2018b; Sr et al., 2018). Formation of a bound-rubber interphase around GO further reduces chain mobility and solvent uptake, reinforcing the barrier response at low loadings (Mensah et al., 2018b).

GO also enhanced rubber–metal adhesion, evaluated via pull-out (shear) bond strength. Figure 18 shows a monotonic increase with filler loading: the bond strength rose from ∼5.4 MPa (pure NBR) to ∼6.7 MPa at 2 phr, an approximately 24% gain (±SD, n = 3). The improvement is attributed to GO’s hydroxyl, carboxyl, and epoxide groups that promote hydrogen bonding and acid–base interactions with metal-oxide layers and typical primers, thereby strengthening the rubber–metal interphase (Mensah et al., 2018b; Salam et al., 2013). Unlike some bulk properties, adhesion continues to benefit even when a fraction of GO aggregates are present because polar clusters located near the interface can still participate in chemical/physical bonding (Salam et al., 2013).

Thermo-oxidative aging performance likewise improved and peaked at 1 phr GO. As summarized in Figures 19c,d, the tensile strength retention f_p_ increased from ∼0.82 (pure) to ∼0.90 at 1 phr, while the elongation retention ε_l_ increased from ∼0.62 to ∼0.65 (±SD, n = 3). These results support GO’s dual role as a heat shield and radical capture agent: stacked sheets hinder oxygen diffusion and heat transfer, while defect/oxygenated sites deactivate radical species generated during thermo-oxidative attack, thereby suppressing chain fragmentation and delaying loss of toughness (Mensah et al., 2018b; Zhong et al., 2018). A slight decline at 2 phr is consistent with microstructural inhomogeneities: initial agglomerates that locally concentrate stress or enhance oxidative pathways. However, the 2 phr GO composite still outperforms pure NBR after aging (Zhong et al., 2018).

The dynamic response followed the same optimum. The recovery elasticity reached ∼12.1% at 1 phr GO (standard rebound test), significantly above pure NBR. This reflects reduced hysteresis: strong filler–rubber interactions constrain viscous relative motion and support elastic energy storage; in dynamic mechanical analysis (DMA), this appears as a depression of the tan δ peak (Mensah et al., 2018a). Together with the swelling and adhesion results, these data indicate that small, well-dispersed quantities of GO are sufficient to reshape interfacial and transport phenomena without leading to significant energy loss.

Overall, ∼1 phr GO yields the optimum balance of properties due to the formation of a well-developed hydrogen-bonded interphase and uniform sheet dispersion. Although 2 phr GO exhibits slightly higher tensile strength, microscopic (AFM/XRD) and dielectric analyses confirm that aggregation begins beyond 1 phr, reducing homogeneity and dielectric stability.

Therefore, 1 phr represents the true performance optimum based on total structure–property synergy rather than a single mechanical parameter. This balance is consistent with the broader literature on graphene-based elastomer nanocomposites, where optimally low loadings outperform higher ones that suffer from aggregation, Payne/Mullins effects, and weaker polymer–filler coupling (Mensah et al., 2018b; Sr et al., 2018). Practically, the results highlight GO as a scalable route to tougher, more durable, and chemically resistant NBR for oil-seal and rubber-to-metal bonded components, provided that loading (∼1 phr) and dispersion/curing conditions are controlled.

## 4 Conclusion

This study provides a new insight into how hydrogen-bonded interphases govern structure–property relationships in low-filler NBR/GO nanocomposites. The critical role of a hydrogen-bonded interphase in governing the structure–property relationships of NBR/GO nanocomposites is elucidated. Through a systematic variation of GO loading (0.5–2.0 phr), it was demonstrated that the optimal macroscopic performance arises from nanoscale interfacial interactions rather than simple filler concentration. At 1 phr GO, FTIR spectroscopy revealed distinct broadening and a slight redshift of the nitrile (C≡N) band, confirming the formation of hydrogen bonds between GO oxygenated groups and NBR nitrile groups. Complementary XRD and AFM analyses verified uniform exfoliation and homogeneous sheet dispersion, while UV-Vis measurements indicated a non-monotonic band gap variation associated with well-dispersed GO domains. These structural and spectroscopic findings correlate directly with the increased tensile strength, tear resistance, rebound resilience, reduced swelling, and superior dielectric stability observed at this composition. Beyond this boundary (≥2 phr GO), partial aggregation of GO nanosheets occurs, which disrupts polymer chain ordering and reduces interfacial homogeneity. As a result, the small increase in tensile strength does not lead to any meaningful improvement in the overall mechanical or dielectric properties. Overall, the results establish that ∼1 phr GO provides the most favorable structure–property synergy by maximizing hydrogen-bonded interphase formation, optimizing dispersion, and maintaining network uniformity. This interphase-controlled reinforcement mechanism offers a low-filler, energy-efficient strategy for designing durable, oil-resistant, and dielectric-stable NBR-based materials suitable for advanced sealing and electronic applications.

## Data Availability

The original contributions presented in the study are included in the article/Supplementary Material; further inquiries can be directed to the corresponding authors.
